# Accuracy of Large Language Models in Answering Dental Examination Questions: A Systematic Review and Meta-Analysis

**DOI:** 10.1016/j.identj.2026.109609

**Published:** 2026-05-18

**Authors:** Mahmood Dashti, Farshad Khosraviani, Atieh Meyari, Mohammad Hosein Amirzade-Iranaq, Akhilanand Chaurasia, Delband Hefzi, Niloofar Ghadimi, Antonin Tichy, Zohaib Khurshid, Falk Schwendicke

**Affiliations:** a Dentofacial Deformities Research Center, Research Institute of Dental Sciences, Shahid Beheshti University of Medical Sciences, Tehran, Iran; b Department of Artificial Intelligence Engineering, Graduate School of Natural and Applied Sciences, Istinye University, Istanbul, Türkiye; c UCLA School of Dentistry, CA, USA; d Department of Esthetic & Restorative Dentistry, School of Dentistry, Tehran Azad University of Medical Sciences, Tehran, Iran; e Universal Scientific Education and Research Network (USERN), Tehran University of Medical Sciences, Tehran, Iran; f Department of Oral Medicine and Radiology, King George’s Medical University, Lucknow, India; g School of Dentistry, Tehran University of Medical Science, Tehran, Iran; h Dental Materials Research Center, TeMS.C., School of Dentistry, Azad University of Medical Sciences, Tehran, Iran; i Clinic for Conservative Dentistry, Periodontology and Digital Dentistry, LMU University Hospital, LMU Munich, Munich, Germany; j Institute of Dental Medicine, First Faculty of Medicine, Charles University, Prague, Czech Republic; k Department of Prosthodontics and Dental Implantology, College of Dentistry, King Faisal University, Al-Ahsa, Saudi Arabia; l Center for Artificial Intelligence and Innovation (CAII), Faculty of Dentistry, Chulalongkorn University, Bangkok, Thailand

**Keywords:** Artificial intelligence, Dental examination, Large language models, meta-analysis

## Abstract

**Introduction:**

Large language models (LLMs), including OpenAI’s GPT family accessed via interfaces such as ChatGPT and Microsoft Copilot, as well as non-GPT systems such as Google Gemini, are increasingly applied in healthcare and dental education. However, the accuracy of these systems in specialized tasks such as answering dental examination questions remains unclear.

**Methods:**

This systematic review and meta-analysis evaluated LLM performance in answering dental questions. Databases searched were PubMed, Embase, Scopus, and Web of Science. Data on question type and number, LLM versions, and accuracy rates were extracted. Pooled accuracy was estimated using a random-effects model; heterogeneity and publication bias were assessed.

**Results:**

A total of 39 studies were included, with ChatGPT-4 being the most frequently evaluated model. The pooled accuracy for LLMs was 63.7% (95% CI: 60.3%-67.1%), with high heterogeneity (*I*² = 91.5%). Subgroup analysis revealed ChatGPT-4 and Copilot (a GPT-based interface) achieved the highest pooled accuracies (∼73% and ∼75%, respectively). Direct comparisons confirmed ChatGPT-4 significantly outperformed earlier versions and some competitor models. Sensitivity analyses supported the robustness of findings.

**Conclusion:**

LLMs demonstrate moderate accuracy in answering dental examination questions and are currently insufficient for autonomous clinical decision-making. When their limitations are explicitly recognized, however, these systems may serve as valuable adjuncts in dental education and examination preparation. Methodological strategies such as structured prompting and retrieval-augmented approaches warrant further investigation but were not the primary focus of the present analysis.

## Introduction

Artificial intelligence (AI) refers to the simulation of human intelligence by machines, enabling them to perform tasks typically requiring human cognitive abilities, such as reasoning, problem-solving, and learning.^1^^,^^2^ Within AI, machine learning (ML) and deep learning represent critical subsets, employing data-driven algorithms to extract patterns, predict outcomes, and make decisions.

Large language models (LLMs) are generative AI systems trained on large text corpora that can interpret prompts and produce human-like text.^3^^,^^4^ In dentistry, LLMs are already being explored for education and assessment support (eg, tutoring, formative feedback, and examination preparation), patient-facing communication (eg, postoperative instructions and informed-consent explanations), evidence-based dentistry tasks (eg, summarizing guidelines and literature), and workflow support (eg, drafting clinical notes and referral letters).^5^^,^^6^ Because dental care is safety-critical and often guideline-driven, their use depends on demonstrable accuracy and reproducibility in dentistry-relevant tasks.

Various LLMs and their associated interfaces are presently available. To avoid conflating models with the user-facing platforms that use them, we list the foundation LLMs first, followed by the major conversational interfaces built on top of them.

Foundation LLMs:

• GPT model family (OpenAI): This includes the models commonly accessed through ChatGPT (eg, GPT-3.5, GPT-4, GPT-4o), which are transformer-based LLMs trained on large-scale corpora and widely applied in education, research, and healthcare.^6^, ^7^, ^8^
• Gemini (Google DeepMind): A multimodal LLM designed for text and image understanding, with strong analytical and scientific reasoning capabilities.^9^
• Claude (Anthropic): A safety-aligned LLM built to minimize hallucinations and improve reliability in sensitive fields such as healthcare.^10^
• LLaMA (Meta): An open-foundation family of LLMs trained on publicly available datasets, supporting transparent and reproducible research.^11^
• DeepSeek LLM: An open-source suite trained on models ranging from 7 billion to 67 billion parameters, using large-scale token datasets and shown to outperform several existing open models on reasoning and coding benchmarks.^12^

Interfaces built on top of foundation LLMs include ChatGPT and Microsoft Copilot (formerly Bing Chat) which are conversational interfaces that provide access to OpenAI’s GPT family models, with Copilot incorporating additional search grounding and safety layers.^3^^,^^6^, ^7^, ^8^ Google’s Gemini interface was formerly released under the name Bard and is powered by the Gemini family of models.^9^ In the present review, model performance is reported according to the platform and model designation used in each original study.

While LLMs can tackle complex queries and offer expert-level responses, their application in dentistry remains an emerging area warranting in-depth evaluation.^6^^,^^10^ LLMs exhibit significant promise in answering dental examination questions, a critical area in both academic and clinical practice. By leveraging extensive training datasets, these models can accurately interpret and respond to multiple-choice questions, case studies, and scenario-based queries. LLMs can support dental education, aid clinical decision-making, perform on standardized exams, and bridge language barriers, enhancing access to dental knowledge.^2^^,^^5^^,^^13^

Despite their potential, questions about accuracy, consistency, and reliability in specialized fields like dentistry persist. Evaluating these models’ performance on dental examinations is thus crucial for validating their applicability and establishing best practices. This systematic review and meta-analysis seeks to evaluate the accuracy and reliability of LLMs in answering dental examination questions. The null hypothesis was that there is no significant variation in accuracy among different LLMs when answering dental examination questions, and that their accuracy does not reach the expected benchmark levels typically required for passing standard dental examination.

## Methods and material

This systematic review and meta-analysis adhered to the guidelines established by the PRISMA (Preferred Reporting Items for Systematic Reviews and Meta-Analyses) criteria.^14^ The process involved the systematic extraction, selection, and screening of relevant research articles.

This review was not registered in PROSPERO, and no protocol was published in advance. We therefore report the full search strategies, prespecified eligibility criteria, screening procedures, and analytical decisions in the Methods and Supplementary materials to support transparency.

We have implemented the PICO framework in accordance with the research question, which was: ‘How accurately can LLMs answer the dental examinations and dental questions?’, to enhance our search strategy:

• Population (P): Dental examination and question sets, including standardized licensing exams, specialty exams, and validated educational question banks in dentistry.
• Intervention (I): Use of LLMs (eg, ChatGPT, Bing AI/Copilot, Google Bard/Gemini, Claude, LLaMA) to answer dental questions.
• Comparison (C): Accuracy against a reference standard (official answer keys, validated question banks, or expert consensus). No consistent direct human comparator across studies.
• Outcome (O): Primary: Accuracy rate (percentage of correct answers). Secondary: (i) between-model/version differences, defined as the absolute difference in accuracy (percentage points) within studies that evaluated two or more LLM models/versions under the same question set; and (ii) subgroup differences in pooled accuracy by LLM model/version group, question type/format, and dental specialty.

## Eligibility criteria

Studies were included if they^1^ evaluated LLMs in answering dentistry-related examination or knowledge-based questions,^2^ provided the model with a defined set of dental questions, and^3^ reported accuracy or performance outcomes.

Studies were excluded if they^1^ were reviews (scoping, narrative, systematic, or meta-analyses),^2^ generated questions using LLMs instead of assessing their ability to answer existing dental questions,^3^ lacked sufficient methodological detail (eg, unspecified model version or missing accuracy data), or^4^ were not focused on dental topics.

We included peer-reviewed full-text articles. Preprints were eligible only if they were indexed in Scopus (identified via the Scopus ‘Secondary documents’ option), had an accessible full text, and reported extractable accuracy outcomes. We did not search non-indexed grey literature sources (eg, websites, blogs, theses) and excluded conference abstracts without sufficient extractable data.

No language restrictions were applied during database searching. However, all studies that met the eligibility criteria and provided extractable accuracy outcomes were available as full texts in English; therefore, all included studies were English-language full texts. A comprehensive overview of the specific search terms employed, the databases accessed, and the number of results that each search yielded is provided in supplementary file 1.

After screening the retrieved records using their titles and abstracts in the first step, we evaluated the full texts of the included records to determine their eligibility. We compiled comprehensive summaries for each study included, detailing their characteristics, methodologies, and relevant outcomes (Table 1).

**Table 1:** Summary of data extracted from the included studies.

N0	Study	Country	Question field	Number of questions	LLM model	Correct answer (percentage) with SD and CI	Source of questions	Question format	Image or text questions
01	Acar^18^	Turkey	Oral surgery	20	ChatGPT-3.5	88.40 ± 0.06	Author-constructed	Open-ended	Text-only
					Microsoft Bing	75.10 ± 0.05			
					Google Bard	70.50 ± 0.02			
02	Ali et al^19^	Qatar	healthcare education	50	ChatGPT-3.5	88%	Author-constructed	Mixed	Text-only
03	Alsayed et al^20^	Saudi Arabia	Oral surgery	15	ChatGPT-4	78%	Author-constructed	Open-ended	Text-only
			Preventive dentistry	15	ChatGPT-4	86%			
			Oral cancer	20	ChatGPT-4	72%			
04	Azadi et al^21^	Iran	Oral surgery	50	Bard	34%	Author-constructed	Mixed	Text-only
					GPT-3.5	36%			
					GPT-4	38%			
					Claude-Instant	38%			
					Microsoft Bing	26%			
05	Batool et al^22^	Pakistan	Operative/Endodontics	40	ChatGPT3.5	60%	Author-constructed	Open-ended	Text-only
			Periodontics	40	ChatGPT3.5	50%			
			Oral Surgery	40	ChatGPT3.5	40%			
			Prosthodontics	40	ChatGPT3.5	60%			
06	Brozovic et al^23^	Croatia	General dentistry	532	Microsoft Bing	71.99%	Mixed/multiple sources	Mix	Text-only
07	Cai et al^24^	China	Oral surgery	30	ChatGPT	100%	Author-constructed	Open-ended	Text-only
08	Chau et al^25^	China	UK and US Dental Licensing exam/Official exam materialinations	1461	ChatGPT 3.5	43.3%	Licensing exam/Official exam material	MCQ	Text-only
					ChatGPT 4	62.7			
09	Danesh et al^26^	Canada	Periodontic	311	ChatGPT3.5	57.9%	Licensing exam/Official exam material	MCQ	Text-only
					ChatGPT4	73.6%			
10	Danesh et al^27^	Canada	General Dentistry	143	ChatGPT3.5	61.3%	Mixed/multiple sources	MCQ	Text-only
					ChatGPT4	76.9%			
11	Daraqel et al^28^	China	Orthodontics	100	ChatGPT3.5	90% (80%-90%, 95%CI)	Author-constructed	Open-ended	Text-only
					Google Bard	80% (80%-90%, 95%CI)			
12	Dashti et al^29^	Iran	INBDE, ADAT, and DAT	122 (knowledge-based)	ChatGPT 3.5	78%	Licensing exam/Official exam material	MCQ	Text-only
					ChatGPT4	88%			
				60 (Case history)	ChatGPT 3.5	70%			
					ChatGPT4	71%			
13	Díaz-Flores García et al^30^	Spain	Endodontics	30	Google Gemini	37.11% ± 2% (34.02%-40.32%, 95%CI)	Author-constructed	Open-ended	Text-only
14	Dursun et al^31^	Turkey	Orthodontics	20	ChatGPT-3.5	83% ± 15%	Author-constructed	Open-ended	Text-only
					ChatGPT-4	90% ± 12.2%			
					Gemini	82% ± 14.4%			
					Copilot	87% ± 16.2%			
15	Farajollahi et al^32^	Iran	Endodontics	100	ChatGPT-3.5	40%	Licensing exam/Official exam material	MCQ	Text-only
16	Freire et al^33^	Spain	Prosthodontics	30	ChatGPT-4	25.6% (22.9%-28.6%, 95%CI)	Author-constructed	Open-ended	Text-only
17	Fuchs et al^34^	Switzerland	Swiss Federal Licensing exam/Official exam materialination in Dental Medicine (SFLEDM)	32	ChatGPT-3	62.6% ± 3.3%	Licensing exam/Official exam material	MCQ	Text-only
					ChatGPT 4	66.7% ± 3.2%			
18	Giannakopoulos et al^35^	Cyprus	General dentistry	20	ChatGPT 4	72% ± 18%	Author-constructed	Open-ended	Text-only
					ChatGPT 3.5	59% ± 24%			
					Bard	57% ± 31%			
					Bing Chat	54% ± 34%			
19	Hatia et al^36^	Italy	Orthodontics	21	ChatGPT 4	81.6%	Author-constructed	Open-ended	Text-only
				7 clinical cases	ChatGPT 4	81.6%			
20	Jacobs et al^37^	USA	Oral surgery	25	ChatGPT 3.5	87.2%	Author-constructed	Open-ended	Text-only
21	Jaworski et al^38^	Poland	Polish Final Dentistry Examination (LDEK)	11 (Clinical case)	ChatGPT-4o	36.36%	Licensing exam/Official exam material	MCQ	Text-only
				188 (Knowledge)		72.87%			
22	Jeong et al^39^	Republic of Korea	General dentistry	16	ChatGPT 3.5	31.3%	Licensing exam/Official exam material	Mixed	Text-only
					ChatGPT 4	93.8%			
					Bard	50.0%			
					Bing	68.8%			
23	Johnson et al^40^	India	Dental trauma	20	Gemini	80%	Author-constructed	Open-ended	Text-only
					Claue AI	80%			
					ChatGPT 3.5	75%			
					Bing	40%			
24	Künzle et al^41^	Germany	Restorative Endodontics	151	ChatGPT-4.o	72%	Question bank	MCQ	Text-only
					Chat-GPT-4	62%			
					ChatGPT-3.5	25%			
					Gemini 1.0	44%			
25	Makrygiannakis et al^42^	Greece	Orthodontics	10	Google’s Bard	4.6/10 46%	Author-constructed	Open-ended	Text-only
					ChatGPT-3.5	3.8/10 38%			
					Chat-GPT-4	4.7/10 47%			
					Microsoft’s Bing	7.1/10 71%			
26	Mohammad-Rahimi et al^43^	USA	Oral pathology, oral medicine, oral radiology	66	Microsoft’s Bing	61.82% ± 17.64%	Author-constructed	Open-ended	Text-only
					ChatGPT3.5	77.48% ± 17.58%			
					ChatGPT4	81.32% ± 16.5%			
					Google Bard	68.48% ± 17.4%			
					Claude	77.08% ± 20.76%			
					Sage	77.98% ± 16%			
27	Mohammad-Rahimi et al^44^	USA	Endodontics	20	ChatGPT-3.5	95%	Author-constructed	Open-ended	Text-only
					Bard	85%			
					Bing	75%			
28	Ohta et al^45^	Japan	Japanese National Dentist Examination	185	ChatGPT 3.5	51.9%	Licensing exam/Official exam material	MCQ	Text-only
					ChatGPT 4	73.5%			
					Bard	66.5%			
29	Ozden et al^46^	Turkey	Dental trauma	25	ChatGPT -3.5	51%	Author-constructed	MCQ (dichotomous)	Text-only
					Bard (Gemini)	64%			
30	Quah et al^47^	Singapore	Oral surgery	259	ChatGPT-3.5	62.2%, 95% CI 56.4%-68.0%	Question bank	MCQ	Text-only
					ChatGPT-4	76.8%, 95% CI 71.4%-82.2%			
					Llama 2	42.5%, 95% CI 37.1%-48.6%			
					Gemini	58.7%, 95% CI 52.9%-64.5%			
					Copilot	72.6%, 95% CI 67.2%-78.0%			
31	Rokhshad et al^48^	USA	Special needs dentistry	25 true/false	ChatGPT 3.5	72% ± 11%	Author-constructed	Open-ended	Text-only
					Google Bard	56%			
					Claude-instant	64% ± 4%			
					Sage	63% ± 8%			
					Llama 2-70b	63% ± 8%			
					Google PaLM	77% ± 5%			
					Claude Instant 100k	69% ± 5%			
					ChatGPT 4	73% ± 2%			
					Claude2 100k	65% ± 2%			
				15 diagnosis	ChatGPT 3.5	35% ± 4%			
					Google Bard	38% ± 4%			
					Claude-instant	35% ± 4%			
					Sage	49% ± 3%			
					Llama 2-70b	33%			
					Google PaLM	33% ± 7%			
					Claude Instant 100k	33%			
					ChatGPT 4	33%			
					Claude2 100k	40% ± 7%			
32	Sabri et al^49^	USA	Periodontology	1312	ChatGPT 4	79.57%	Licensing exam/Official exam material	MCQ	Text-only
					ChatGPT 3.5	64.93%			
					Gemini	72.86%			
33	Song et al^50^	South Korea	Korean National Dental Hygienist Examination (2019-2023)	777	ChatGPT 4	74.2%	Licensing exam/Official exam material	MCQ	Text-only
					ChatGPT 3.5	61.3%			
					Gemini	61.7%			
34	Suárez et al^51^	Spain	Endodontic	60	ChatGPT-4	57.33% ± 0.82% (55.72% -58.95%, 95%CI)	Author-constructed	MCQ (dichotomous)	Text-only
35	Suárez et al^52^	Spain	Oral surgery	30	ChatGPT-4	71.7% (68.9 –74.6%, 95%CI)	Mixed/multiple sources	Open-ended	Not reported
36	Turunç Oğuzman et al^53^	Turkey	Dental specialty Entrance exam in Turkey	Prosthodontics 126	ChatGPT 3.5	35.7%	Licensing exam/Official exam material	MCQ	Text-only
					Bard	38.9%			
				Radiology 123	ChatGPT 3.5	52.8%			
					Bard	52.8%			
37	Vaira et al^54^	Italy	Oral Surgery	72 Open-ended	ChatGPT 4	81.7%	Author-constructed	Mixed	Text-only
				72 True/False	ChatGPT 4	84.7%			
38	Yamaguchi et al^55^	Japan	Japanese National Dental Hygienist Examination	73	ChatGPT 3.5	63.0%	Licensing exam/Official exam material	MCQ	Text-only
					ChatGPT 4	75.3%			
					Bard	66.7%			
					Bing Chat	68.5%			
39	Yunsun et al^56^	South Korea	Pediatric	32	ChatGPT 3.5	35.3% ± 5.6%	Licensing exam/Official exam material	MCQ	Text-only
					Gemini	33.0% ± 4.0%			

CI, confidence interval; SD, standard deviation; Not reported, indicates the primary study did not explicitly describe the item source or format.

## Research strategy and screening

Four databases were searched: MEDLINE via PubMed, Embase, Scopus, and Web of Science. Searches were conducted within the native interfaces/platforms of PubMed, Embase, Scopus, and Web of Science. Within Scopus, we additionally applied the ‘Secondary documents’ option to capture additional indexed record types, which may include items originating from preprint servers (eg, medRxiv) when indexed in Scopus. We did not conduct a separate search directly within preprint repositories. All retrieved records were exported to a reference manager for deduplication prior to screening, Deduplication was then performed using automated matching (DOI, title, and author) followed by manual verification of uncertain matches, and only the deduplicated set proceeded to title/abstract screening, the search conducted in January 2025 using the queries listed in Supplementary Table 1. Titles and abstracts were independently screened by D.H. and M.H.A., with disagreements resolved by M.D. The same two reviewers (D.H. and M.H.A.) also performed the full-text assessment and data extraction independently, and any discrepancies were again adjudicated by M.D. Only studies meeting all eligibility criteria and providing complete data were included in the final analysis. Deduplication was performed using automated matching (DOI, title, and author) followed by manual verification of uncertain matches. After deduplication, each unique record was screened once to prevent double counting across databases.

**Fig. 1:**
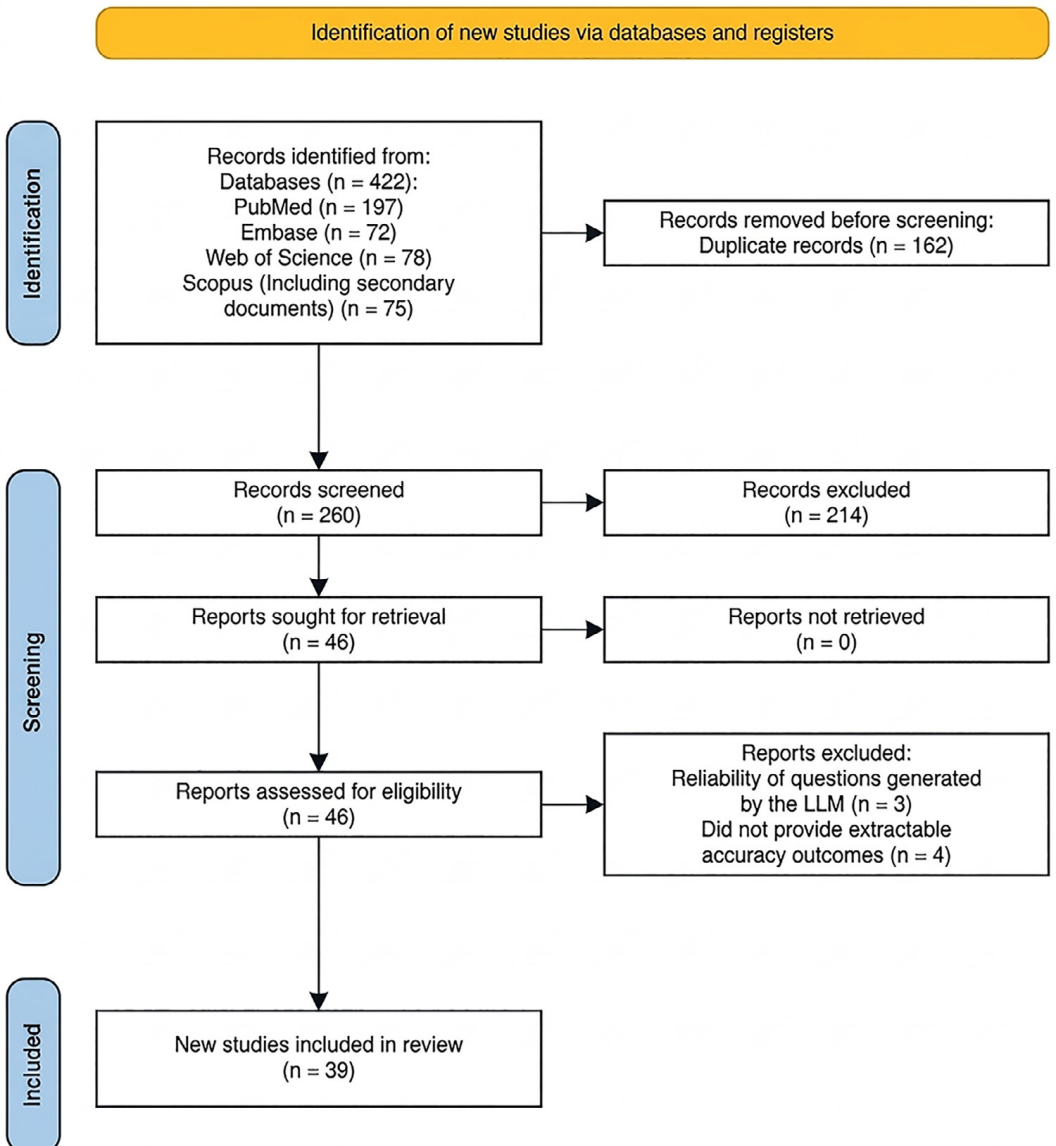
PRISMA flow diagram of study selection.

The data extracted from the included articles are summarized in Table 1. These include author details, publication year, origin country, question type, number of questions, LLM model, and accuracy of correctly answered questions. A PRISMA flow chart, created using R software,^15^ was produced to illustrate the selection process (Figure 1).

### Quality evaluation

Risk of bias and applicability concerns were assessed using a modified QUADAS-2 framework appropriate for studies evaluating large language models on question-answering tasks. Two reviewers (D.H. and M.H.A.) independently evaluated all included studies using predefined signalling questions, with disagreements resolved by a third reviewer (M.D.). Each domain was judged as low, high, or unclear risk of bias, with unclear ratings assigned when reporting was insufficient. Full domain-level judgments and the complete signalling-question checklist are provided in Supplementary Table 2.

**Domain mapping and signalling questions**

• Domain 1: Item selection and sampling of questions
  Signalling questions: Were questions sourced from official examinations or validated question banks? Were inclusion and exclusion criteria for items prespecified? Was any selective or post-hoc inclusion likely to inflate accuracy?
  Applicability focus: Does the question pool represent the intended dental examination domain?
• Domain 2: Index test procedures for LLM response generation and scoring
  Signalling questions: Were prompts, temperature, and model versions prespecified and applied consistently? Were raters blinded to the reference standard during scoring? Were tie-break and partial-credit rules defined in advance?
  Applicability focus: Do the prompting and evaluation conditions reflect realistic examination use?
• Domain 3: Reference standard
  Signalling questions: Was the reference based on an official answer key or expert consensus? Was the reference determined independently of the LLM output? Were discrepancies resolved through a documented adjudication process?
  Applicability focus: Does the reference standard accurately represent correct dental knowledge?
• Domain 4: Flow and timing
  Signalling questions: Were all items included in the analysis? Was the test order consistent across models? Were repeated queries, model updates, or use of external tools controlled or reported?
  Applicability focus: Does the test flow reflect a stable and unbiased evaluation process?

### Summary measures and data synthesis

A meta-analysis was conducted using data from the included studies to determine the proportion of correct answers for each LLM. In cases where publications evaluated multiple AI models, each model was analysed individually and designated by the model version and author name.

To avoid conflating brand names with underlying models, we harmonized labels across studies. Microsoft Copilot (formerly called ‘Bing Chat/AI’ during our time window of evaluation) is an interface powered by OpenAI GPT with Microsoft safety/policy layers; we analyse it as Copilot (GPT family interface). Google Bard was rebranded to Gemini; when studies reported Bard during the Bard to Gemini transition, we classified them under Gemini (Bard-era). Where a paper explicitly evaluated ChatGPT-4 within OpenAI’s ChatGPT product, we label it ChatGPT-4 (OpenAI interface). These decisions preserve UI/prompting differences without mislabelling Copilot as a distinct base model.

Statistical analyses were performed using R 4.4.1 and RStudio, with the ‘meta, metafor, metasens, dplyr, and ggplot2’ packages. A random-effects model using the Inverse variance method with Freeman-Tukey double arcsine transformation was employed for the primary analysis. Heterogeneity was assessed using *I*² statistics, with values of 25%, 50%, and 75% indicating low, moderate, and high heterogeneity, respectively. Publication bias was evaluated using funnel plots and Egger’s test.^16^ Subgroup analyses on different LLM models were performed to explore potential sources of heterogeneity. To ensure uniformity, proportion values expressed as percentages in the studies were transformed into decimal format.

### Sensitivity analyses

Robustness was assessed through several methods, including leave-one-out influence analysis, alternative estimators such as DerSimonian-Laird and maximum likelihood, comparison of fixed-effect models, and Baujat diagnostic plots to identify influential studies.^17^ The fixed-effect model was used only as a sensitivity check to evaluate whether the pooled estimate materially changed under the assumption of a common true effect, and not as the primary model because substantial clinical and methodological heterogeneity was expected.

## Results

The search yielded 422 records. After removing duplicates, 260 records remained for title and abstract screening, of which 214 were excluded. The remaining 46 reports were assessed in full text; 7 reports were excluded (3 evaluated LLM-generated question reliability rather than answering existing questions, and 4 did not report extractable accuracy outcomes). Finally, 39 studies were included in the data extraction and meta-analysis (Figure 1). Reasons for exclusion included three articles that evaluated the reliability of questions generated by the LLM and four articles that did not provide accuracy measures for the LLM.

All 39 included studies were used in the qualitative synthesis. These studies spanned a broad geographic distribution, and the top publishing countries included the USA (*n* = 5), Turkey (*n* = 4), and Spain (*n* = 4). The overwhelming majority of the included studies (*n* = 38, 97.4%) examined one of OpenAI’s ChatGPT models (versions 3.5 and/or 4), with ChatGPT being examined in a total of 68 instances across the studies. Other LLMs evaluated included Gemini (including studies originally referring to Bard, *n* = 16), Claude (*n* = 9), Copilot (GPT family interface, *n* = 7), among others.

The primary objective across most studies was to evaluate the models’ performance in answering a range of dental questions. Questions relating to oral surgery were used in 9 studies, followed by general questions from national dental examinations (*n* = 8 studies), and endodontics (*n* = 5). The question format varied from multiple-choice and true/false questions to open-ended and clinical scenario-based questions. The mean number of questions posed was 85.2 (standard deviation (SD) =143.2) with a range of 7 to 1461. Outcomes were typically reported as percentages of correct responses, with some studies also providing SD, confidence intervals (CI), or inter-model comparisons (Table 1).

The 39 included studies used heterogeneous question sources and formats. Question provenance most commonly consisted of author-constructed question sets (*n* = 21/39, 53.8%), followed by licensing or official examination materials (*n* = 13/39, 33.3%). A smaller proportion used question banks (*n* = 2/39, 5.1%) or mixed/multiple sources (*n* = 3/39, 7.7%). Regarding question format, studies used multiple-choice questions (MCQ, including dichotomous items) in 17/39 studies (43.6%), open-ended formats in 17/39 (43.6%), and mixed formats in 5/39 (12.8%). Almost all studies evaluated text-only questions (38/39, 97.4%), while image involvement was not reported in one study (1/39, 2.6%). These variations represent plausible contributors to between-study heterogeneity and were therefore captured in Table 1 for transparent interpretation of pooled estimates. These variables were reported descriptively to characterize heterogeneity; they were not used as prespecified moderators in meta-regression due to inconsistent reporting across studies.

### Risk of bias assessment

Under the item selection and sampling domain, 22 of the 39 included studies (56.4%) were judged at high risk of bias, primarily due to the use of author-generated or non-standardized question sets rather than official or validated examination items. While such question sources may introduce subjectivity and reduce comparability across studies, they consistently aimed to assess the same construct, namely the accuracy of LLMs in answering dental knowledge questions, and were therefore considered unlikely to alter the overall direction of the pooled results.

All studies (100%) were rated as low risk in the index test domain, reflecting clear documentation of prompting procedures, independent scoring, and reproducible conditions. The reference standard domain was low risk in 92.3% (36/39) of studies that used official answer keys or expert consensus; three studies (7.7%) had unclear risk because of insufficient reporting. The flow and timing domain was low risk in all studies, with complete inclusion of evaluated items and no evidence of selective omission.

Regarding applicability concerns, most studies were rated low concern across domains. The adapted ‘item selection’ domain showed moderate concern in studies using locally constructed question banks, as these may not fully represent standardized dental curricula. The index test and reference standard domains showed low concern, indicating that the evaluated models and scoring criteria were appropriate to the intended research question. Overall, despite variability in question sources, methodological consistency and transparent reporting across studies support the robustness and applicability of the meta-analytic findings (Supplementary Table 2).

## Meta-analysis

### Pooled effect size estimates

**Fig. 2:**
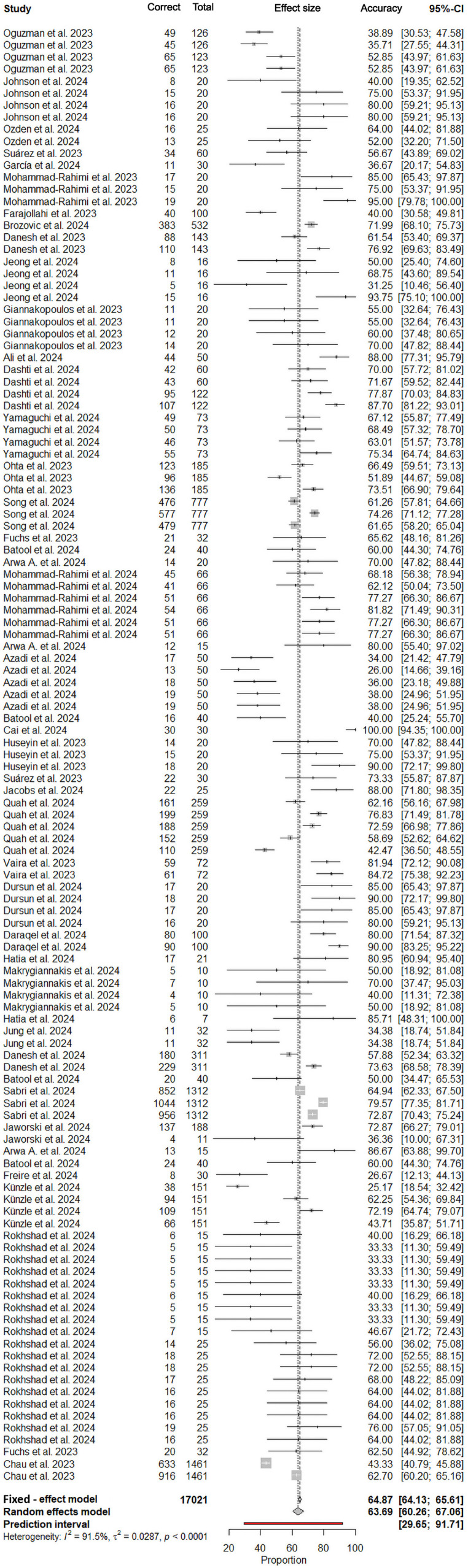
Forest plot of the overall pooled accuracy of large language models in answering dental examination questions.

Under the random-effects model, the meta-analysis of 39 studies yielded an overall pooled answer rate of 63.69% (95% CI: 60.26%-67.06%), with substantial between-study heterogeneity (*I*² = 91.5%, τ² = 0.0287; *P* < .0001). Prediction intervals spanning 29.65% to 91.71% highlight the variability in expected correct answer rate across future implementations. Sensitivity analyses comparing fixed- and random-effects models revealed similar pooled estimates, with fixed-effects results at 64.87% (95% CI: 64.13%-65.61%) (Figure 2).

### Subgroup analysis by LLM model

**Fig. 3:**
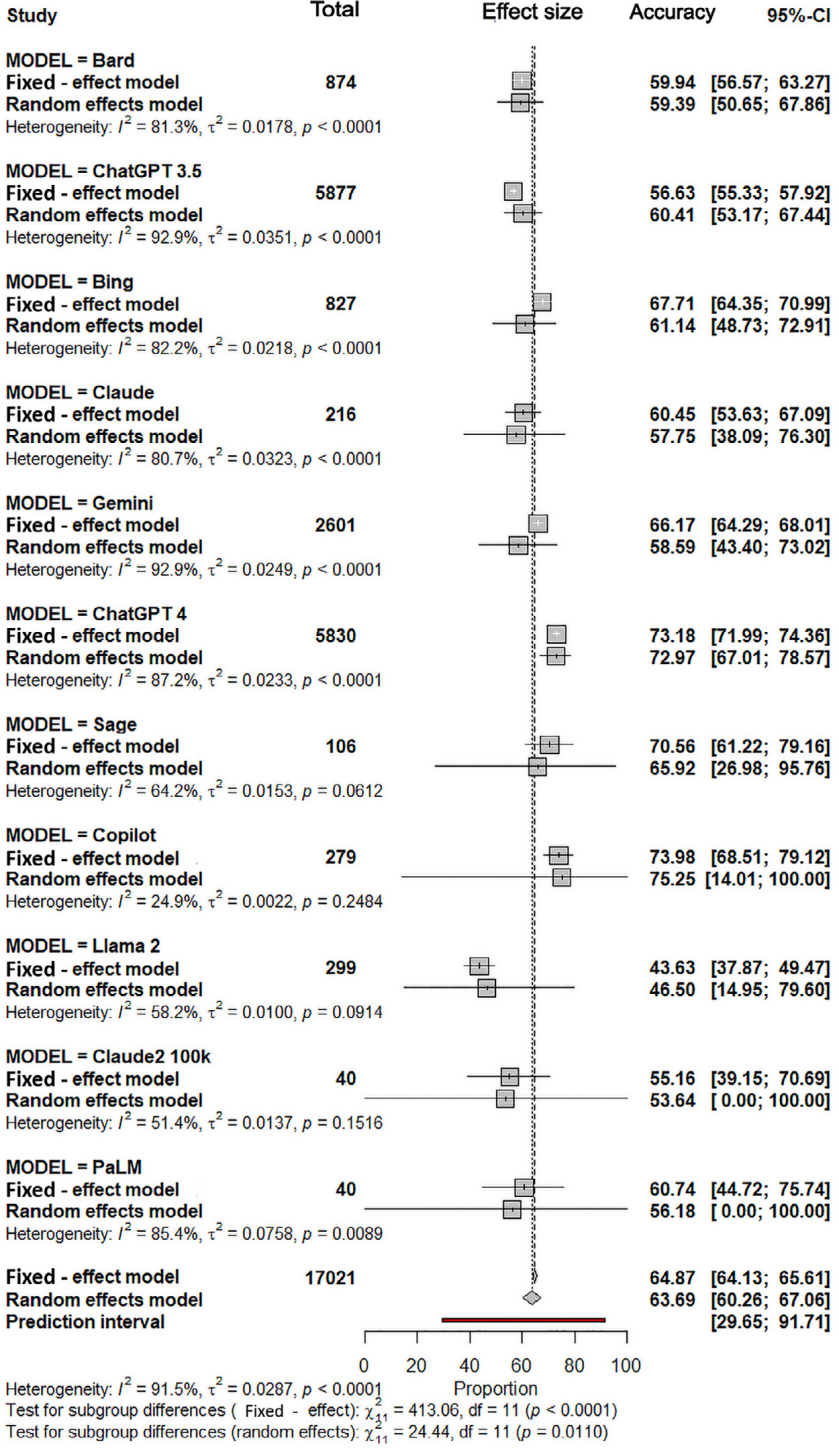
Forest plot of subgroup analysis according to large language model type.

**Fig. 4:**
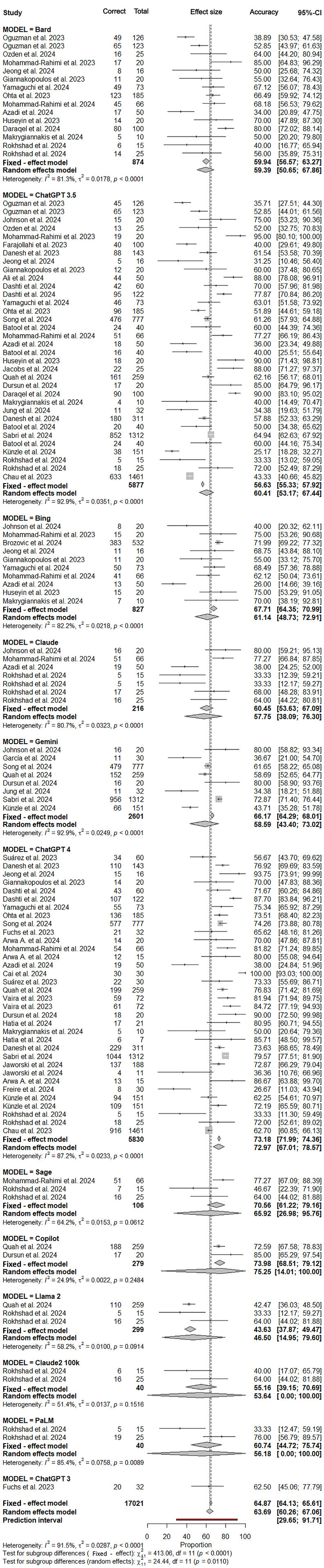
Forest plot of individual study estimates grouped by large language model subgroup.

Subgroup analyses stratified by architecture demonstrated statistically significant differences in performance (Q-between = 24.44, *P* = .011). Copilot (GPT family interface) and ChatGPT-4 (OpenAI interface) achieved the highest pooled estimates (Copilot: 75.25%; 95% CI: 14.01%-100%; ChatGPT4: 72.97%; 95% CI: 67.01%-78.57%), with limited heterogeneity (I^2^=24.9%, *P* = .25) for Copilot and higher heterogeneity (*I*² = 87.2%, *P* < .0001) for ChatGPT. Overall, 34 studies employed these two LLM-based chatbots/interfaces. For the majority of comparisons of accuracy, significant differences between different LLMs were identified (Fig. 3, Fig. 4).

### Publication bias

Quantitative assessments of publication bias yielded mixed evidence. While Egger’s regression test indicated no small-study effects (*P* = .56), Begg’s test results (*P* = .31) suggested consistency with funnel plot asymmetry. Trim-and-fill analysis imputed six hypothetical missing studies, adjusting the pooled accuracy to 65.43% (95% CI: 61.88%-68.89%), ie, very similar to the base-case analysis.

### Sensitivity analysis

**Fig. 5:**
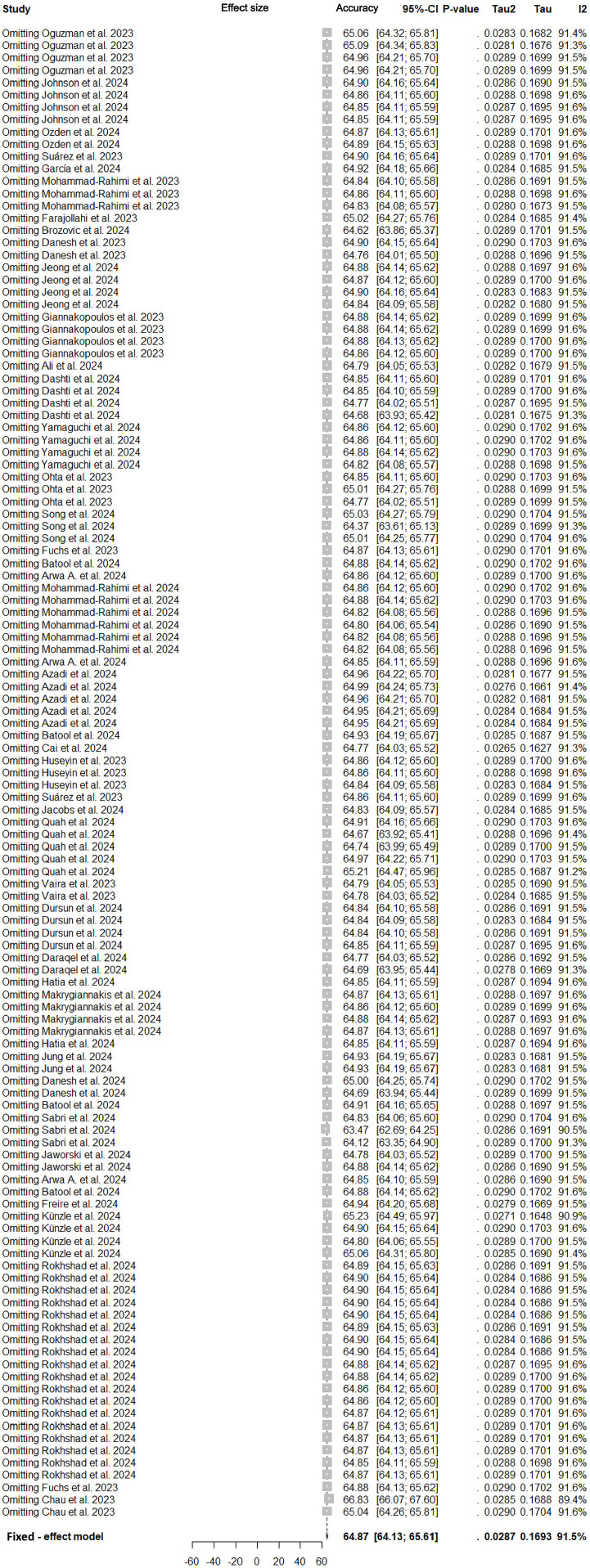
Leave-one-out sensitivity analysis of pooled accuracy.

Methodological robustness was confirmed through multiple sensitivity checks. Leave-one-out analysis demonstrated stable pooled accuracy estimates ranging from 64.13% to 65.66%, with the most influential study (Chau et al^25^) altering results by 1.96% upon exclusion (Figure 5).

## Discussion

Since its launch in November 2022, ChatGPT has gained widespread popularity given its ease of use, accessibility, and ability to automate various cognitive tasks in a time-efficient manner. Ever since, ChatGPT and several other LLM-based tools have become increasingly adopted across many fields, including medical and dental sciences.^6^^,^^57^^,^^58^ Twelve of the included studies were published in 2023, compared with 27 in 2024. This reflects the great interest these LLMs have garnered in the dental community since their launch, a trend expected to persist as LLMs advance and new developments are tested for dental use. Notably, two studies also investigated LLMs’ accuracy in answering dental hygienist examination questions, highlighting that these models can benefit other professionals in oral healthcare.^50^^,^^55^

Implementations vary from diagnosis and decision-making to education and research.^59^^,^^60^ The accuracy of the models’ output has been a topic of debate, with numerous researchers designing studies to evaluate LLMs’ performance against standardized examination questions.^31^^,^^39^^,^^44^^,^^61^ In detail, the accuracy of LLMs in tackling medical examination questions has been previously systematically reviewed.^61^ However, that is not the case for the dental field. The present study bridges this knowledge gap by synthesizing a meta-analytic estimate of the accuracy of contemporary LLMs in answering dental questions. As of January 2025, the results showed that OpenAI’s ChatGPT was the most widely examined model, with ChatGPT-4 being the most accurate among those evaluated. The pooled accuracy of the LLMs amounted to 63.69% (95% CI: 60.26%-67.06%). Because Copilot is a GPT–powered interface rather than a distinct base LLM, its slightly higher accuracy likely reflects interface-level differences (guardrails, context windows, search/grounding, default prompting).

The results of the present study are in line with a previous systematic review investigating LLMs’ accuracy in responding to medical questions,^62^ which concluded that ChatGPT was the most widely used and most accurate model, particularly in diagnosis and classification tasks.^62^ Furthermore, ChatGPT-4 outperformed version 3.5, achieving accuracies above 90% in some instances.^31^^,^^39^^,^^44^ This is comparable to recent studies that reported accuracies of up to 100% in diagnosing clinical cases.^63^^,^^64^ However, in some of these evaluations, the questions and their correct answers may have been publicly available, which introduces the possibility that such content was included in the training data of the evaluated LLMs, potentially inflating performance estimates. Notably, the LLMs demonstrated superior performance when answering dental questions compared to medical questions, with a recent systematic review reporting an accuracy of 56%.^65^ Nonetheless, the models showed inconsistencies. Results varied widely, with some models scoring as low as 25%,^41^ which is in agreement with previous lower-range accuracy estimates of 18.3%.^64^

The pooled accuracy of 63.69% falls within the statistically acceptable range but may be unacceptable for reliable use in clinical practice, where patient safety could be compromised by inaccurate LLM recommendations. Furthermore, because these models are trained on data available only up to a certain point, they may not always reflect the most recent clinical practice guidelines, nor are they necessarily able to comprehensively assess a clinical case scenario. Some newer systems offer optional online search or retrieval mechanisms that can supplement their training data, but this only partially mitigates these limitations. Therefore, such models should not be relied upon for real-world clinical decision-making, but may offer value as supporting tools in dental education or as an aid in preparing for dental examinations, provided their limitations are clearly recognized. As noted by Hager et al,^66^ current LLMs fail to consistently follow diagnostic or treatment guidelines across pathologies and cannot reliably interpret laboratory results, which underscores their unsuitability for autonomous clinical decision-making. In dentistry specifically, Giannakopoulos et al^35^ warn that imprudent use of LLMs may adversely impact patient care. Additionally, given the potential of LLMs to produce misinformation,^67^ they currently cannot replace human knowledge and skill.^68^

Our review also differs from prior dentistry-focused syntheses that concentrated specifically on dental licensing examinations. For example, Liu et al^69^ evaluated LLM performance in dental licensing examinations worldwide. In contrast, we used broader eligibility criteria that included licensing examinations, specialty board-style assessments, validated educational question banks, and dentistry-relevant knowledge assessments across multiple subdisciplines. We also separated model/version and interface labels (eg, Copilot as a GPT-based interface), incorporated a QUADAS-2–based risk-of-bias assessment tailored to LLM question-answering studies, and performed model-stratified pooling with multiple sensitivity diagnostics to test robustness.

Our pooled estimates suggest that LLM performance in dentistry has been evaluated mainly in controlled, text-based knowledge settings, most commonly using multiple-choice questions with a defined answer key. This format resembles exam preparation and foundational knowledge checks rather than real-world clinical decision-making. Consequently, the observed accuracy should not be interpreted as evidence that LLMs can safely perform independent clinical reasoning, treatment planning, or documentation without expert oversight.

A key finding is the substantial heterogeneity across studies, which is plausibly driven by differences in question format (multiple-choice versus open-ended), question provenance (official licensing examinations and validated banks versus author-constructed sets), and whether items were scenario-based. In particular, author-constructed question sets can systematically influence measured accuracy depending on topic selection, wording, and difficulty distribution. This aligns with our risk-of-bias assessment, where item selection and reporting practices were common concerns. These issues likely explain why pooled accuracy varies across studies even for the same named model family.

From an educational perspective, the results support cautious use of LLMs as supplementary learning tools (eg, generating explanations, highlighting key concepts, and supporting formative self-testing) while emphasizing that correctness must be verified, especially in high-stakes domains such as oral pathology/oncology, pharmacology, and medical emergencies. The strongest near-term value is therefore likely in education and structured knowledge support, rather than autonomous clinical recommendation. Our findings complement prior work focused on dental licensing examinations^69^ by including a broader range of dentistry-relevant assessments and by explicitly examining heterogeneity sources across study designs. Broader evidence from licensing-exam evaluations in health professions similarly emphasizes variability by question type and the need for careful governance and oversight.^70^

Jin et al^71^ also pooled ChatGPT-3.5 and GPT-4 performance across national health licensing examinations (medicine, pharmacy, dentistry, nursing) and reported an overall accuracy around 70%, with GPT-4 consistently outperforming ChatGPT-3.5. Dentistry constituted only a small portion of their included evidence and the review was restricted to multiple-choice licensing items. In contrast, our review is dentistry-focused and includes broader question sources and formats, reinforcing that reported accuracy is highly context-dependent and should not be generalized across tasks or model releases.

Training data transparency and other ethical issues remain concerns with these LLMs.^72^ Recent work has highlighted that dental applications raise additional governance challenges, including data provenance, privacy, fairness, explainability, and responsibility during clinical implementation, underscoring the need for structured ethical frameworks when deploying LLMs in practice.^72^

While it appears that some dental-related content was used in their training, it is not clear on which data they were trained. This makes it problematic to countercheck the reliability of LLMs’ answers, as they may be based on inaccurate or outdated information and perpetuate biases in the training data.^73^, ^74^, ^75^ After all, these models function as sophisticated pattern recognition systems and do not generate new knowledge nor critically assess data before synthesizing an output. Nevertheless, recent evidence suggests that fine-tuning LLMs with carefully curated resources and using contextual prompting can significantly improve their accuracy in answering specialized dental examination questions.^76^ The inherent variability in dental literature and clinical practice guidelines means that model outputs reflect the biases and inconsistencies present in their training data. The limited explainability of models’ output is a relatively common issue with many AI applications,^77^ making it difficult to assess the reasoning behind their specific answers or recommendations.

This review has several limitations that should be acknowledged. Substantial heterogeneity was observed across the included studies, reflecting variability in question sources, formats, dental specialties, model versions, and evaluation procedures. High heterogeneity is common in accuracy and prevalence meta-analyses; notably, a recent methodological review reported a median *I*² value of 96.9% across 134 meta-analyses,^78^ underscoring that elevated heterogeneity alone does not invalidate pooled estimates when robustness is supported by sensitivity analyses, as observed in the present study. Limitations of the evidence base include the exclusion of non-English-language full texts, which may reflect language or indexing bias, and that most included studies evaluated textbook-style or examination-based questions rather than complex real-world clinical scenarios, which limits generalizability to routine clinical practice. In addition, incomplete reporting of model parameters, platform versions, and query conditions in several studies constrained reproducibility and risk-of-bias assessment. Finally, a further limitation is the absence of prospective protocol registration, which may reduce transparency and increase the risk of selective reporting despite our detailed reporting of methods and analyses.

It is also noteworthy that the rapid pace of LLM updates is an important interpretive constraint. Most primary studies assessed specific, time-stamped versions (commonly GPT-3.5 and GPT-4) because those were the publicly accessible models during their study periods. Our meta-analysis therefore estimates performance of the versions evaluated in the published literature up to 31 January 2025, rather than claiming that the same accuracy applies to newer model releases. Ongoing model updates may change performance; periodic (or ‘living’) updates of this review will be necessary as new dentistry-specific evaluations of newer models become available.

Taken together, these findings indicate that while current LLMs demonstrate moderate accuracy in dental knowledge assessments, they are not suitable for autonomous clinical decision-making. When their limitations are explicitly recognized, however, LLMs may serve as useful adjuncts in dental education and examination preparation, provided that outputs are interpreted critically and under professional supervision.

## Conclusion

This systematic review and meta-analysis demonstrates that large language models currently achieve moderate accuracy when answering dental examination questions. Although newer GPT-4–class systems generally outperform earlier versions, overall performance remains insufficient for unsupervised clinical application. Given the precision required for dental diagnosis and treatment planning, LLMs should not be relied upon as autonomous decision-making tools. When their methodological and conceptual limitations are appropriately acknowledged, however, these systems may offer meaningful value as supportive resources in dental education and examination preparation. Future research should prioritize standardized evaluation protocols, transparent reporting of model configurations, and systematic investigation of performance-enhancing strategies such as structured contextual prompting and retrieval-augmented approaches to better define the appropriate role of LLMs in dentistry.

## Author contributions

M.D.: Conception and design of study, Acquisition of data, Drafting of article and/or critical revision, Final approval of manuscript. F.Kh.: Conception and designed of study, Final approval of manuscript, Analysis of data. A.M.: Acquisition of data, Analysis of data, Drafting of article and/or critical revision, Final approval of manuscript. M.A.: Conception and design of study, Analysis of data, Drafting of article and/or critical revision, Final approval of manuscript. A.Ch.: Conception and design of study, Acquisition of data, Drafting of article and/or critical revision, Final approval of manuscript. D.H.: Analysis of data, Drafting of article and/or critical revision, Final approval of manuscript. N.Gh.: Acquisition of data, Analysis of data, Drafting of article and/or critical revision, Final approval of manuscript. A.T.: Final approval of manuscript, Drafting of article and/or critical revision. Z.Kh.: Conception and design of study, Drafting of article and/or critical revision, Final approval of manuscript. F.Sch.: Final approval of manuscript, Drafting of article and/or critical revision. All authors have critically reviewed and approved the final draft and are responsible for the content and similarity index of the manuscript.

## Funding

This research did not receive any specific grant from funding agencies in the public, commercial, or not-for-profit sectors.

## Declaration of competing interest

The authors declare the following financial interests/personal relationships which may be considered as potential competing interests:

The author is an Editorial Board Member for this journal and was not involved in the editorial review or the decision to publish this article. Given his role as editorial board member, had no involvement in the peer review of this article and had no access to information regarding its peer review. Full responsibility for the editorial process for this article was delegated to another journal editor. If there are other authors, they declare that they have no known competing financial interests or personal relationships that could have appeared to influence the work reported in this paper.

## Appendix Supplementary materials

Image, application 1

Image, application 2
