# Supplementary material for: Accuracy of Large Language Models in Answering Dental Examination Questions: A Systematic Review and Meta-Analysis
Source: Int Dent J. 2026 May 18;76(4):109609. doi: 10.1016/j.identj.2026.109609 (PMC13202568; doi:10.1016/j.identj.2026.109609)
Supplement: Supplementary file 1 [file mmc1.docx]

Supplementary Table 01**:** Full search queries for each database.

| Database | Search query | Results |
| --- | --- | --- |
| Pubmed | ("ChatGPT" OR "Chat GPT" OR "Chat-GPT" OR "LLM" OR "LLMs" OR "Bard" OR "Large Language Models" OR "Large Language Model") AND ("Orthodontics" OR "Endodontics" OR "Oral Pathology" OR "Oral Radiology" OR "Pediatric Dentistry" OR "Periodontics" OR "Prosthodontics" OR "Dentistry Public Health" OR "Oral Surgery" OR "Oral and Maxillofacial Surgery" OR "dental exam" OR "dental question" OR "dentistry exam" OR "dentistry questions" OR "dentistry question" OR "dental questions") | 197 |
| Embase | ('chatgpt'/exp OR 'chatgpt' OR 'chat gpt'/exp OR 'chat gpt' OR 'chat-gpt' OR 'llm' OR 'llms' OR 'bard'/exp OR 'bard' OR 'large language models' OR 'large language model'/exp OR 'large language model') AND (('orthodontics'/exp OR orthodontics OR 'endodontics'/exp OR endodontics OR 'mouth disease'/exp OR 'mouth disease' OR 'oral pathology'/exp OR 'oral pathology' OR 'oral radiology' OR 'pediatric dentistry'/exp OR 'pediatric dentistry' OR 'periodontics'/exp OR periodontics OR 'prosthodontics'/exp OR prosthodontics OR 'dentistry public health'/exp OR 'dentistry public health' OR 'oral surgery'/exp OR 'oral surgery' OR oral) AND ('maxillofacial surgery'/exp OR 'maxillofacial surgery') OR 'oral and maxillofacial surgery' OR 'dental exam'/exp OR 'dental exam' OR 'dental question' OR 'dentistry exam' OR 'dentistry questions' OR 'dentistry question' OR 'dental questions') | 72 |
| Scopus | ( TITLE-ABS-KEY ( "ChatGPT" ) OR TITLE-ABS-KEY ( "Chat GPT" ) OR TITLE-ABS-KEY ( "LLM" ) OR TITLE-ABS-KEY ( "LLMs" ) OR TITLE-ABS-KEY ( "Bard" ) OR TITLE-ABS-KEY ( "Large Language Models" ) OR TITLE-ABS-KEY ( "Large Language Model" ) OR TITLE-ABS-KEY ( "Chat-GPT" ) ) AND ( TITLE-ABS-KEY ( "Orthodontics" ) OR TITLE-ABS-KEY ( "Endodontics" ) OR TITLE-ABS-KEY ( "Oral Pathology" ) OR TITLE-ABS-KEY ( "Oral Radiology" ) OR TITLE-ABS-KEY ( "Pediatric Dentistry" ) OR TITLE-ABS-KEY ( "Periodontics" ) OR TITLE-ABS-KEY ( "Prosthodontics" ) OR TITLE-ABS-KEY ( "Dentistry Public Health" ) OR TITLE-ABS-KEY ( "Oral Surgery" ) OR TITLE-ABS-KEY ( "Oral and Maxillofacial Surgery" ) OR TITLE-ABS-KEY ( "dental exam" ) OR TITLE-ABS-KEY ( "dental question" ) OR TITLE-ABS-KEY ( "dentistry exam" ) OR TITLE-ABS-KEY ( "dentistry questions" ) OR TITLE-ABS-KEY ( "dentistry question" ) OR TITLE-ABS-KEY ( "dental questions" ) ) | 70 |
| Scopus Secondary | ( TITLE-ABS-KEY ( "ChatGPT" ) OR TITLE-ABS-KEY ( "Chat GPT" ) OR TITLE-ABS-KEY ( "LLM" ) OR TITLE-ABS-KEY ( "LLMs" ) OR TITLE-ABS-KEY ( "Bard" ) OR TITLE-ABS-KEY ( "Large Language Models" ) OR TITLE-ABS-KEY ( "Large Language Model" ) OR TITLE-ABS-KEY ( "Chat-GPT" ) ) AND ( TITLE-ABS-KEY ( "Orthodontics" ) OR TITLE-ABS-KEY ( "Endodontics" ) OR TITLE-ABS-KEY ( "Oral Pathology" ) OR TITLE-ABS-KEY ( "Oral Radiology" ) OR TITLE-ABS-KEY ( "Pediatric Dentistry" ) OR TITLE-ABS-KEY ( "Periodontics" ) OR TITLE-ABS-KEY ( "Prosthodontics" ) OR TITLE-ABS-KEY ( "Dentistry Public Health" ) OR TITLE-ABS-KEY ( "Oral Surgery" ) OR TITLE-ABS-KEY ( "Oral and Maxillofacial Surgery" ) OR TITLE-ABS-KEY ( "dental exam" ) OR TITLE-ABS-KEY ( "dental question" ) OR TITLE-ABS-KEY ( "dentistry exam" ) OR TITLE-ABS-KEY ( "dentistry questions" ) OR TITLE-ABS-KEY ( "dentistry question" ) OR TITLE-ABS-KEY ( "dental questions" ) ) | 5 |
| Web of Science | (TS=("ChatGPT" OR "Chat GPT" OR “Chat-GPT” OR "LLM" OR "LLMs" OR "Bard" OR "Large Language Models" OR "Large Language Model")) AND TS=("Orthodontics" OR "Endodontics" OR "Oral Pathology" OR "Oral Radiology" OR "Pediatric Dentistry" OR "Periodontics" OR "Prosthodontics" OR "Dentistry Public Health" OR "Oral Surgery" OR "Oral and Maxillofacial Surgery" OR "dental exam" OR "dental question" OR "dentistry exam" OR "dentistry questions" OR "dentistry question" OR "dental questions") | 78 |
