# Supplementary material for: Accuracy of Large Language Models in Answering Dental Examination Questions: A Systematic Review and Meta-Analysis
Source: Int Dent J. 2026 May 18;76(4):109609. doi: 10.1016/j.identj.2026.109609 (PMC13202568; doi:10.1016/j.identj.2026.109609)
Supplement: Supplementary file 2 [file mmc2.docx]

Supplementary Table 02: Results of the risk of bias assessment using a modified QUADAS-2 tool.

|  | Authors (year) | Risk of bias | | | | Applicability concerns | | |
| --- | --- | --- | --- | --- | --- | --- | --- | --- |
|  |  | Item selection and sampling of questions | Index test | Reference standard | Flow and timing | Item selection | Index test | Reference standard |
|  | Acar, 2023 (18) | High | Low | Unclear | Low | High | Low | Low |
|  | Ali et al. ,2024 (19) | High | Low | Low | Low | High | Low | Low |
|  | Alsayed et al.,2024 (20) | Low | Low | Low | Low | Low | Low | Low |
|  | Azadi et al. ,2024 (21) | Low | Low | Low | Low | Low | Low | Low |
|  | Batool et al. ,2024 (22) | Low | Low | Low | Low | Low | Low | Low |
|  | Brozovic et al.,2024 (23) | Low | Low | Low | Low | Low | Low | Low |
|  | Cai et al. ,2024 (24) | High | Low | Unclear | Low | High | Low | Low |
|  | Chau et al. ,2023 (25) | Low | Low | Low | Low | Low | Low | Low |
|  | Danesh et al. ,2024 (26) | Low | Low | Low | Low | Low | Low | Low |
|  | Danesh et al. ,2023 (27) | Low | Low | Low | Low | Low | Low | Low |
|  | Daraqel et al. ,2024 (28) | High | Low | Low | Low | High | Low | Low |
|  | Dashti et al. ,2024 (29) | Low | Low | Low | Low | Low | Low | Low |
|  | Díaz-Flores García et al. ,2024 (30) | High | Low | Low | Low | High | Low | Low |
|  | Dursun et al. ,2024 (31 | High | Low | Low | Low | High | Low | Low |
|  | Farajollahi et al.,2023 (32) | Low | Low | Low | Low | Low | Low | Low |
|  | Freire et al. ,2024 (33) | High | Low | Low | Low | High | Low | Low |
|  | Fuchs et al. ,2023 (34) | Low | Low | Low | Low | Low | Low | Low |
|  | Giannakopoulos et al.,2023 (35) | High | Low | Low | Low | High | Low | Low |
|  | Hatia et al.,2024 (36) | High | Low | Low | Low | High | Low | Low |
|  | Jacobs et al. ,2024 (37) | High | Low | Low | Low | High | Low | Low |
|  | Jaworski et al. ,2024 (38) | Low | Low | Low | Low | Low | Low | Low |
|  | Jeong et al. ,2024 (39) | High | Low | Low | Low | High | Low | Low |
|  | Johnson et al. ,2024 (40) | High | Low | Low | Low | High | Low | Low |
|  | Künzle et al. ,2024 (41) | Low | Low | Low | Low | Low | Low | Low |
|  | Makrygiannakis et al. ,2024 (42) | High | Low | Low | Low | High | Low | Low |
|  | Mohammad-Rahimi et al. ,2024 (43) | High | Low | Low | Low | High | Low | Low |
|  | Mohammad-Rahimi et al. ,2023 (44) | High | Low | Low | Low | High | Low | Low |
|  | Ohta et al., 2023 (45) | Low | Low | Low | Low | Low | Low | Low |
|  | Ozden et al.,2024 (46) | High | Low | Low | Low | High | Low | Low |
|  | Quah et al. ,2024 (47) | High | Low | Low | Low | High | Low | Low |
|  | Rokhshad et al.,2024 (48) | High | Low | Low | Low | High | Low | Low |
|  | Sabri et al. ,2024 (49) | Low | Low | Low | Low | Low | Low | Low |
|  | Song et al,2024 (50) | Low | Low | Low | Low | Low | Low | Low |
|  | Suárez et al. ,2023 (51) | High | Low | Low | Low | High | Low | Low |
|  | Suárez et al. ,2023 (52) | High | Low | Low | Low | High | Low | Low |
|  | Turunç Oğuzman et al,2023 (53) | High | Low | Low | Low | High | Low | Low |
|  | Vaira et al.,2023 (54) | High | Low | Low | Low | High | Low | Low |
|  | Yamaguchi et al. ,2024 (55) | Low | Low | Low | Low | Low | Low | Low |
|  | Yunsun et al., 2024 (56) | High | Low | Low | Low | High | Low | Low |
